# Dysfunctional Inhibitory Mechanisms in Locus Coeruleus Neurons of the Wistar Kyoto Rat

**DOI:** 10.1093/ijnp/pyu122

**Published:** 2015-03-04

**Authors:** C Bruzos-Cidón, N Llamosas, L Ugedo, M Torrecilla

**Affiliations:** Department of Pharmacology, Faculty of Medicine and Dentistry, University of the Basque Country UPV/EHU, 48940 Leioa, Spain (Drs Bruzos-Cidón, Ugedo, and Torrecilla, and Llamosas).

**Keywords:** depression, GABA, glutamate, patch-clamp

## Abstract

**Background:**

The noradrenergic nucleus *locus coeruleus* (LC) has functional relevance in several psychopathologies such as stress, anxiety, and depression. In addition to glutamatergic and GABAergic synaptic inputs, the activation of somatodendritic α_2_-adrenoceptors is the main responsible for LC activity regulation. The Wistar Kyoto (WKY) rat exhibits depressive- and anxiety-like behaviors and hyperresponse to stressors. Thus, the goal of the present study was to investigate *in vitro* the sensitivity of α_2_-adrenoceptors, as well as the glutamatergic and GABAergic synaptic activity on LC neurons of the WKY strain.

**Methods:**

For that purpose patch-clamp whole-cell recordings were done in LC slices.

**Results:**

The α_2_-adrenoceptors of LC neurons from WKY rats were less sensitive to the effect induced by the agonist UK 14 304 as compared to that recorded in the Wistar (Wis) control strain. In addition, the GABAergic input to LC neurons of WKY rats was significantly modified compared to that in Wis rats, since the amplitude of spontaneous GABAergic postsynaptic currents was reduced and the half-width increased. On the contrary, no significant alterations were detected regarding glutamatergic input to LC neurons between rat strains.

**Conclusions:**

These results point out that in WKY rats the inhibitory control exerted by α_2_-adrenoceptors and GABAergic input onto LC neurons is dysregulated. Overall, this study supports in this animal model the hypothesis that claims an imbalance between the glutamatergic-GABAergic systems as a key factor in the pathophysiology of depression.

## Introduction

Animal models, despite inherent limitations, are critical to achieve substantial progress in understanding the neurobiological bases of psychiatric disorders (Nestler and Hyman, 2010; Overstreet, 2012; Armario and Nadal, 2013). Among these models, the Wistar Kyoto (WKY) rat is frequently used to study the pathophysiology of depression. A substantial amount of work has shown that WKY rats present innate depressive-like behavior in different tests, such as the forced swimming test and the learned helplessness test (Pare, 1989; Pare and Redei, 1993; Lahmame and Armario, 1996; Pare and Tejani-Butt, 1996; Lahmame et al., 1997; Lopez-Rubalcava and Lucki, 2000; Rittenhouse et al., 2002; Tejani-Butt et al., 2003; Will et al., 2003; Malkesman, Braw, et al., 2006; Carr et al., 2010; Yamada et al., 2013; Kyeremanteng et al., 2014; Nam et al., 2014). They also exhibit disturbances in the sleep-wake cycle, including increased rapid-eye-movement sleep and sleep fragmentation (Dugovic et al., 2000), which are prevalent symptoms in depressive patients (Bunney and Bunney, 2013). In addition, hormonal abnormalities of the hypothalamic-pituitary-adrenal axis, often disrupted in depressive patients, have been extensively reported in this rat strain as compared to the Wistar (Wis) control strain (Pare and Redei, 1993; Redei et al., 2001; Solberg et al., 2001; Rittenhouse et al., 2002; De La Garza and Mahoney, 2004; Malkesman, Maayan, et al., 2006). Acute noradrenergic response to stress—measured by tyrosine hydroxylase mRNA levels, noradrenaline (NA) release, or c-fos stimulation—is also attenuated in WKY rats compared to Sprague-Dawley rats (Pardon et al., 2002). By contrast, chronic stress results in a greater sensitization of the hypothalamic-pituitary-adrenal axis and central noradrenergic system in WKY as compared to Sprague-Dawley rats (Morilak et al., 2005). A recent electrophysiological study shows that neuronal basal firing rate of the *locus coeruleus* (LC) nucleus is augmented and the sensitivity of α_2_-adrenoceptors reduced in WKY as compared to Wis rats (Bruzos-Cidon et al., 2014).

The LC is the main source of NA in most brain structures, including cortical and limbic regions. It has an important role in mediating behavioral states such as mood, motivation, stress, attention, and arousal (see for review Sara and Bouret, 2012). In fact, this brain structure has been proposed as a part of the central stress circuitry, which has functional relevance in the pathophysiology of several mental disorders, including stress, anxiety, and depression (Itoi and Sugimoto, 2010). The LC basal activity is mainly regulated by the activation of somatodendritic α_2_-adrenoceptors, which open a G protein–coupled inwardly-rectifying potassium (GIRK) channel leading to a decrease of cell excitability *in vivo* and *in vitro* (Cedarbaum and Aghajanian, 1976; Williams et al., 1985; Aghajanian and Wang, 1986; Torrecilla et al., 2008, 2013). Additionally, major glutamatergic input from the paragigantocellularis nucleus influences LC activity (Aston-Jones et al., 1991), acting on postsynaptically located NMDA (Olpe et al., 1989) and non-NMDA receptors (Williams et al., 1991). Glutamate may also be synthesized and released within the LC neurons themselves (Noriega et al., 2007). The inhibitory input to the LC is provided by GABAergic neurons from the nucleus prepositus hypoglossi (Aston-Jones et al., 1991) and a population of small GABAergic interneurons (Iijima and Ohtomo, 1988; Aston-Jones et al., 2004), acting onto pre- and postsynaptically located GABA_A_ receptors (Corteen et al., 2011). Interestingly, dysfunctional glutamatergic and GABAergic systems are also considered to play a relevant role in the pathophysiology of depression (Krystal et al., 2002; Sanacora et al., 2012).

Given the role of the LC in the development of mental disorders, we have investigated the neurophysiology of this brain area in the WKY strain. For that purpose, whole-cell voltage-clamp recordings were carried out in LC brain slices to evaluate the sensitivity of α_2-_adrenoceptors, as well as the glutamatergic and GABAergic synaptic activity of this noradrenergic nucleus in the WKY rat. As a control group we used the Wis strain, from which WKY rats were derived (Okamoto and Aoki, 1963).

## Experimental Procedures

### Animals

Male Wis and WKY rats weighing 175–250g were provided by Harlan Interfauna Ibérica. This weight range ensures the use of age-matched animals, according to the animal supplier. Every effort was made to minimize the suffering of the animals and to use the minimum number of animals possible. Experimental protocols were reviewed and approved by the Local Committee for Animal Experimentation at the University of the Basque Country. All the experiments were performed in compliance with the European Community Council Directive on the Protection of Animals Used for Experimental and Other Scientific Purposes (86/609/EEC) and with the Spanish Law (RD 1201/2005) for the care and use of laboratory animals.

### Drugs

(2S)-3-[(1S)-1-(3,4-Dichlorophenyl )ethyl]amino-2-hydroxypropyl](phenylmethyl) phosphinic acid hydrochloride (CGP 55845 hydrochloride), D-(-)-2-Amino-5-phosphonopentanoic acid (D-AP5), 6,7-Dinitroquinoxaline-2,3-dione disodium salt (DNQX disodium salt), 4-Hydroxyquinoline-2-carboxylic acid (kynurenic acid), (5S,10R)-(+)-5-Methyl-10,11-dihydro 5H-dibenzo[a,d] cyclohepten-5,10-imine maleate [(+)-MK-801 maleate], picrotoxin, and 17-Hydroxyyohimban-16-carboxylic acid methyl ester hydrochloride (yohimbine hydrochloride) were obtained from Tocris Bioscience. Chloral hydrate and 5-Bromo-6-(2-imidazolin-2-ylamino) quinoxaline (UK 14 304) were obtained from Sigma-Aldrich and (S)-1-Aminopropane-1,3-dicarboxylic acid (glutamate) from Research Biochemicals International.

Drug stocks, except chloral hydrate, were prepared in distilled water and diluted in artificial cerebrospinal fluid (ACSF) right before use. Chloral hydrate was prepared in 0.9% saline and applied by one intraperitoneal injection to achieve animal anaesthesia.

### Patch-Clamp Recordings of Locus Coeruleus Neurons from Rat Brain Slices

Slice preparation and whole-cell patch-clamp recordings were performed as described by Torrecilla et al. (2008).

#### Brain Slice Preparation 

Animals were anaesthetized with chloral hydrate (400mg/kg, i.p) and decapitated. The brain was immediately extracted and placed in cooled ACSF containing (in mM): 125 NaCl, 2.5 KCl, 1.2 MgCl_2_, 26 NaH_2_CO_3_, 1.25 NaH_2_PO_4_, 2.4 CaCl_2_, and 11 D-glucose saturated with 95% O_2_ and 5% CO_2_ (pH 7.3–7.4). In order to reduce neuronal damage, (+)-MK-801 maleate (10 µM) was added to ACSF. To record excitatory synaptic activity, kynurenic acid (100 µM) was substituted for (+)-MK-801 maleate. Horizontal brainstem sections (220 μm) containing the LC were cut using a vibrotome (VT1200S; Leica Microsystems). LC slices were incubated in warmed (35ºC) ACSF for at least 30min before recording.

#### Neuronal Identification and Recording 

The slice mounted on a recording chamber was maintained at 35–37 ºC and perfused with ACSF at a flow rate of 1.5–2ml/min, through bath perfusion by gravity. LC neurons were visualized, using an upright microscope with infrared optics (Eclipse E600FN, Nikon), as a dense and compact group of cells located on the lateral border of the central gray and the fourth ventricle, just anterior to the genu of the facial nucleus (Figure 1A).

Electrophysiological parameters, UK 14 304, and glutamate-induced whole-cell currents, as well as spontaneous glutamatergic and GABAergic postsynaptic currents (sEPSC and sIPSC, respectively) and evoked glutamatergic and GABAergic postsynaptic currents (eEPSC and eIPSC, respectively) were recorded in voltage-clamp mode. For that purpose, glass pipettes (2–4 MΏ) prepared from borosilicate glass capillaries (World Precision Instruments) with a micropipette puller (PC-10, Narishige CO., LTD) were filled with an internal solution containing (in mM): 130 K-Gluconate, 5 NaCl, 1 MgCl_2_, 1 ethylene glycol-bis(2-aminoethylether)-N,N,N’,N’-tetraacetic acid (EGTA), 10 4-(2-hydroxyethyl)piperazine-1-ethanesulfonic acid (HEPES), 2 adenosine 5’-triphosphate magnesium salt (Mg-ATP), 0.5 guanosine 5’-triphosphate sodium salt hydrate (Na-GTP), and 10 phosphocreatine disodium salt hydrate (pH: 7.4, 280 mOsm). Currents were recorded with the membrane potential held at -50 mV. LC neurons were identified by the presence of a resting inwardly-rectifying potassium (IRK) conductance by stepping the membrane potential from -40 to -120 mV in -10 mV increments (100ms/step; Figure 1B; Williams et al., 1988). The term inward rectification refers to an increase in slope of the current-voltage (I/V) relationship near the reversal potential (Nernst prediction for potassium, -105 mV for our recording conditions). Immediately after neuron identification membrane capacitance (Cm), series resistance and input resistance (Rm) were measured using Clampex software. In some experiments, resting membrane potential (Vm) was also recorded (I = 0). Two concentrations of UK (0.15 µM and 1.5 µM) were administered to calculate UK ratio. For that purpose, the peak current induced by 0.15 µM UK was divided by the peak current induced by 1.5 µM UK. In addition, activation of GIRK currents by UK were detected using the above described I/V protocol before and during drug application. Activation of G-protein coupled receptors opens a GIRK conductance, evidenced by an increase in the resting I/V (Williams et al., 1988). The amplitude of UK-induced currents in LC neurons was also normalized to the capacitance of the neuron, resulting in a current density. Series resistance was monitored throughout the experiment and the cell was discarded if it exceeded 15 MΩ.

To evaluate sEPSCs and eEPSCs, voltage-clamp recordings were performed with 100 µM picrotoxin in the ACSF to block GABA_A_ receptors. The glass pipettes were filled with an internal solution containing (in mM): 70 CsSO_4_, 20 CsCl, 20 NaCl, 1.5 MgCl_2_, 5 HEPES, 1 EGTA, 2 Mg ATP, and 0.5 Na-GTP (pH: 7.4, 280 mOsm). To trigger both α-Amino-3-hydroxy-5-methyl-4-isoxazolepropionic acid (AMPA) and NMDA receptors-mediated responses, eEPSCs were recorded at a holding potential of +40 mV. By passing constant current pulses (15–50 µA, at every 20 s, using the Iso-Flex stimulus isolator (A.M.P.I)) through a glass pipette situated into the LC and filled with external solution, 30 eEPSCs were evoked. After events stabilization, NMDA receptors antagonist D-AP5 (100 µM) was applied in order to isolate AMPA receptors-mediated eEPSCs. Glutamatergic total response and AMPA receptors-mediated eEPSCs were calculated from the average of at least seven consecutive events. NMDA receptors-mediated eEPSCs were calculated by subtraction of AMPA receptors-mediated response to total eEPSCs. Peak currents of isolated AMPA and NMDA receptors-mediated eEPSC were used to calculate the AMPA/NMDA ratio.

To record sIPSCs and eIPSCs, we added 1 μM CGP55845, 50 µM D-AP5 and 20 µM DNQX to the ACSF to block GABA_B,_ NMDA and AMPA/kainate receptors, respectively. The internal solution contained (in mM): 115 KCl, 20 NaCl, 1.5 MgCl_2_, 5 HEPES, 10 1,2-bis(2-aminophenoxy)ethane-N,N,N’,N’-tetraacetic acid (BAPTA), 2 Mg-ATP, and 0.5 Na-GTP (pH: 7.4, 280 mOsm). eIPSCs were recorded at a holding potential of -50 mV and one (1–10 mA) or five pulse trains (1–10 mA) were trigged at 10 Hz using a bipolar tungsten electrode situated posterior to the LC. GABA_A_ receptors-mediated eIPSCs were calculated by averaging at least seven consecutive events. Changes in the probability of GABA release were investigated by measuring the IPSCn/IPSC 1 ratio. To do that the amplitude of a consecutive IPSC (IPSCn) was divided by the amplitude of the first IPSC (IPSC1).

### Data Acquisition and Analysis

Recordings were detected with an Axopatch-200B (Axon Instruments), filtered at 5kHz and digitized with a Digidata 1322A (Axon Instruments). Data were sampled at 10kHz and analyzed with Clampex 10.2 software (Axon Instruments).

Recordings were filtered post hoc at 1kHz and visually inspected to select manually synaptic events. Template detection was used to select spontaneous synaptic currents (Clampfit 10.3 software), with deflections <7 pA excluded from analysis. The template was generated from an average of multiple spontaneous events, the selection was fitted to the 3.5 threshold of the template, and each peak was visually inspected. The duration of synaptic events was determined by measuring the width at 50% of the peak amplitude (half-width). In all cases, spontaneous activity from each neuron was measured for 1 minute. All data were extracted using Clampfit 10.3 software.

Graph Prism (v.5.01; GraphPad Software, Inc.) was used for statistical evaluations. Non-repeated two-way ANOVA was used to compare the outward current induced by two different concentrations of UK in both rat strains, Wis and WKY. The amplitude of spontaneous synaptic currents was analyzed by the Kolmogorov-Smirnov test (Clampfit 10.3 software). The rest of the electrophysiological parameters evaluated in the present work were statistically compared through strains using Student’s *t*-test. The level of significance was considered as *p* < 0.05.

## Results

### Electrophysiological Findings

#### Intrinsic Electrophysiological Properties of the Locus Coeruleus Neurons from Wistar Kyoto Rats 

To study whether LC neurons from WKY and Wis rats had substantial differences in their intrinsic electrical properties, besides visual localization, we first identify LC neurons by the presence of a resting IRK conductance (see in Experimental Procedures, the Neuronal Identification section and Figure 1B). A total of 138 LC neurons were recorded: 65 neurons from the Wis strain (n = 12 animals) and 73 neurons from the WKY strain (n = 12 animals). The basal properties of these LC cells, such as Cm, Rm, and Vm, did not statistically differ between strains (Table 1).

**Figure:**
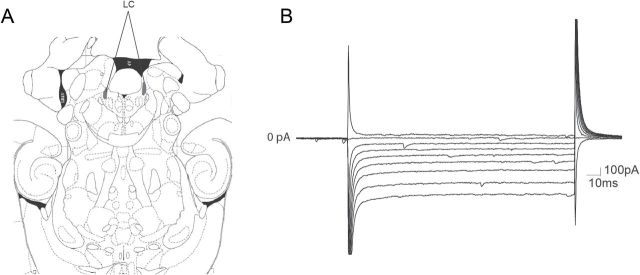
Figure 1. Localization and electrophysiological identification of noradrenergic *locus coeruleus* (LC) neurons *in vitro*. (A) Representative picture of LC localization in the horizontal plane (Paxinos and Watson, 1997). (B) Representative traces of inwardly-rectifying potassium currents in a LC neuron triggered by stepping the membrane potential in -10 mV increments for 100ms.

**Table 1.** *In vitro* intrinsic electrophysiological properties of *locus coeruleus* neurons recorded in Wistar and Wistar Kyoto rats.

	Cm (pF)	Rm (MΩ)	Vm (mV)
Wistar	142.9±6.6	260.8±13.7	-56.38±2.81
Wistar Kyoto	153.0±7.5	237.8±12.2	-52.14±1.72

All values represent the mean ± standard error of the mean of experiments. To calculate Cm and Rm, Wis n = 65 and WKY n = 74. For Vm Wis n = 13 and WKY n = 25.

No statistical significances were found between strains using unpaired two-tailed *t*-tests.

Cm, membrane capacitance; Rm, membrane resistance; Vm, resting membrane potential.

#### *In Vitro* Noradrenergic Transmission in Locus Coeruleus Neurons of Wistar Kyoto Rats 

**Figure 2.**
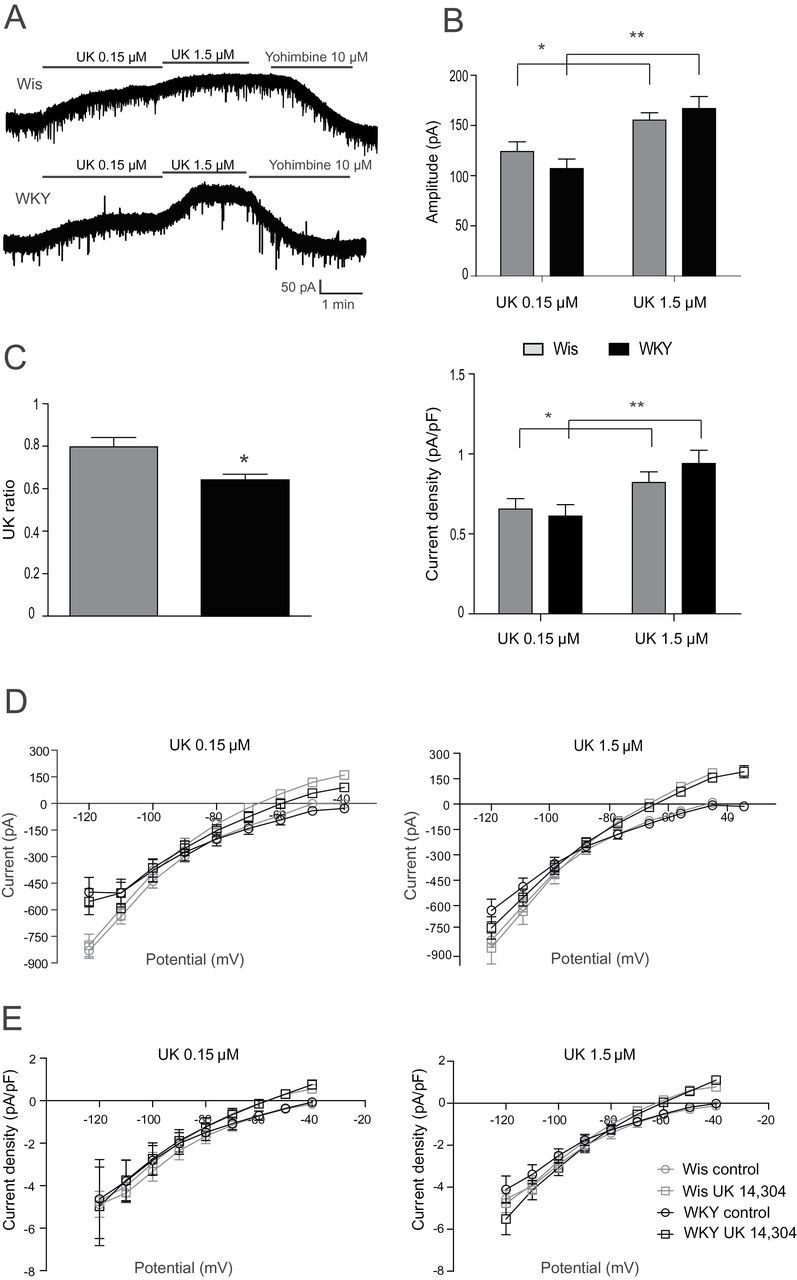
Whole-cell currents induced by UK 14 304 in *locus coeruleus* (LC) neurons of Wistar (Wis) and Wistar Kyoto (WKY) rats. (A) Representative recordings of the outward current induced by UK (0.15µM and 1.5µM) in LC neurons of Wis and WKY rats. Administration of the α_2_-antagonist yohimbine (10 µM) completely reversed the UK-induced current. (B) Summary bar graphs displaying the mean amplitudes of the UK-induced outward currents and mean of current densities in a concentration-dependent manner (*p < 0.01 and **p < 0.001, unpaired two-tailed *t*-test). (C) Summary data of UK ratio (*p = 0.028, unpaired two-tailed *t*-test). (D) Whole-cell current-voltage relationship in LC neurons of Wis (grey symbols) and WKY (black symbols) rats at rest (circles) and during (squares) UK perfusion (n = 6–7). (E) Current-voltage relationship in LC neurons of Wis (grey symbols) and WKY (black symbols) rats for the data concerning basal (circles) and induced current densities (pA/pF) during (squares) UK perfusion (n = 6–7).

To evaluate the noradrenergic transmission on LC neurons from Wis and WKY strains, we recorded the whole-currents induced by two different concentrations of the α_2_-adrenoceptor agonist UK (0.15 µM and 1.5 µM). In LC neurons of both Wis and WKY rats, 0.15 µM and 1.5 µM UK application caused a concentration-dependent outward current at a holding potential of -50 mV (*p* < 0.001, two-way ANOVA; Figure 2A and B). The amplitude of the peak current induced by each concentration was not statistically different between strains (0.15 µM UK, Wis: 124.9±7.79 pA, n = 7 and WKY: 110.1±9.02 pA n = 5; 1.5 µM UK, Wis: 152.1.9±7.61 pA, n = 7 and WKY: 154.9±14.52 pA, n = 5; Figure 2B, upper panel). Accordingly, the current densities induced by UK perfusion were not different between Wis and WKY rats (0.15 µM UK, Wis: 0.65±0.06 pA/pF, n = 7 and WKY: 0.61±0.07 pA/pF, n = 5; 1.5 µM UK, Wis: 0.82±0.06 pA/pF, n = 7 and WKY: 0.94±0.08 pA/pF, n = 5; Figure 2B, lower panel). However, when we analyzed the UK ratio of the peak current induced by both concentrations of UK, data showed that LC neurons from WKY rats were significantly less sensitive than those from the Wis strain (WKY: 0.64±0.03, n = 5; Wis: 0.79±0.04, n = 7; *p* = 0.028, unpaired two-tailed *t*-test; Figure 2C). The UK-induced outward current was completely reversed by α_2_-adrenoceptor antagonist yohimbine (10 µM; Figure 2A).

To further characterize the agonist-induced outward current in the LC neurons, I/V relationships were obtained before and during UK perfusion, by hyperpolarizing the membrane potential (see Experimental Procedures). While bath perfusion of 0.15 µM UK increased the I/V relationship recorded in LC neurons of Wis rats to a greater extent than that in WKY rats (Figure 2D), perfusion of 1.5 µM UK induced a similar increment in both strains (Figure 2D). The reversal potential of the I/V obtained during UK perfusion was similar to that predicted by Nernst for a potassium conductance (-105 mV) (Williams et al., 1988). Current densities of the basal and UK-induced I/V were also provided, and were all similar in both strains (Figure 2E).

#### Study of the Glutamatergic Input to Locus Coeruleus Neurons of Wistar Kyoto Rats 

**Figure 3.**
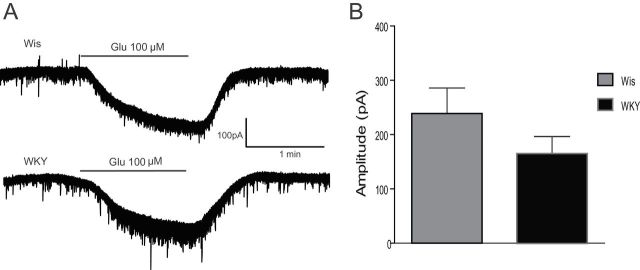
Whole-cell currents induced by glutamate in *locus coeruleus* (LC) neurons of Wistar (Wis) and Wistar Kyoto (WKY) rats. (A) Representative traces and (B) summary data of the mean amplitude of the inward current induced by a brief application of glutamate (100 µM) in LC neurons of Wis and WKY rats. The holding potential was -50 mV. Bars represent the mean ± standard error of the mean of experiments (n = 5–6).

Bath perfusion of glutamate (100 µM) onto LC neurons induced an inward current of similar amplitude in both Wis and WKY strains (238.1±47.66 pA, n = 6; 164.4±32.00 pA, n = 5, respectively; Figure 3A and B).

**Figure 4.**
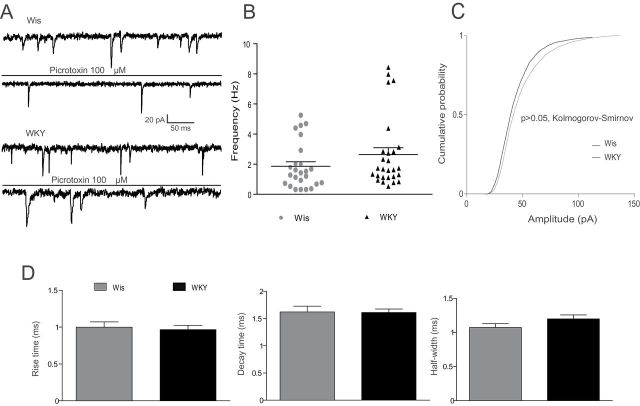
Spontaneous excitatory input to *locus coeruleus* (LC) neurons in Wistar (Wis) and Wistar Kyoto (WKY) rats. (A) Representative current traces of spontaneous glutamatergic postsynaptic currents (sEPSCs) before and after picrotoxin perfusion. (B) Dot graphs showing the frequency of sEPSCs in LC neurons of Wis and WKY rats. (C) Cumulative histogram of sEPSCs amplitude showing no differences between Wis and WKY rats. (D) Bar graphs showing rise time, decay time, and half-width of sEPSCs recorded on LC neurons from Wis (n = 16) and WKY (n = 23) rats. Bars represent the mean ± standard error of the mean of experiments.

Next, we examined the spontaneous glutamatergic synaptic input to LC neurons in the presence of picrotoxin (100 µM) to ensure the blockade of GABA_A_ receptors (Figure 4A). Thus, frequency (Figure 4B) and amplitude (Figure 4C) of sEPSCs measured in LC neurons were indistinguishable between Wis (1.86±0.31 Hz and 47.52±0.38 pA, n = 25) and WKY rats (2.64±0.46 Hz and 41.32±0.23 pA, n = 27). Kinetic of the sEPSCs was also evaluated by measuring their rise and decay time and half-width parameter, and showed no differences between strains (Figure 4D).

**Figure 5.**
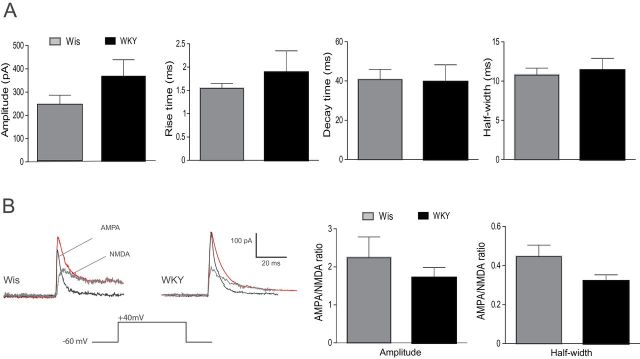
Evoked glutamatergic synaptic activity in *locus coeruleus* (LC) noradrenergic neurons of Wistar and Wistar Kyoto rats. (A) Bar graphs showing rise time, decay time and half-width of evoked glutamatergic postsynaptic currents (eEPSCs) recorded in LC neurons of Wis (n = 8) and WKY (n = 12) rats. (B) Illustration of α-Amino-3-hydroxy-5-methyl-4-isoxazolepropionic acid (AMPA) and NMDA receptors mediated eEPSC at holding potential of +40 mV on LC neurons of Wis and WKY rats. Bar graphs showing the amplitude and half-width of the AMPA/NMDA ratio recorded from both rat strains (n = 7–10).

Secondly, glutamate release was evoked by electrical single stimulus (5–15 µA) at a holding potential of +40 mV to simultaneously trigger both AMPA and NMDA receptor-dependent eEPSCs (Figure 5B). No differences were observed in the eEPSC amplitudes (Wis: 236.9±44.16 pA, n = 8; WKY: 359.5±73.46 pA, n = 12) nor in the parameters associated with neurotransmitter kynetics (rise time for Wis: 1.54±0.10ms, n = 8 and WKY: 1.88±0.46ms, n = 10; decay time for Wis: 40.63±5.30ms, n = 8 and WKY: 39.61±8.60ms, n = 10; half-width for Wis: 10.77±0.9ms, n = 8 and WKY: 11.37±1.55ms, n = 12; Figure 5A). In addition, we further analyzed possible strain differences regarding the roles of AMPA and NMDA glutamatergic receptors on the excitatory activity of LC neurons by calculating the AMPA/NMDA ratio in each rat strain (see Experimental Procedures). Both Wis and WKY rats showed similar AMPA/NMDA ratios regarding the amplitude and the half-width of eEPSC in LC neurons (amplitude for Wis: 2.24±0.55, n = 7 and WKY: 1.71±0.27, n = 10; half-width for Wis: 0.44±0.05, n = 7 and WKY: 0.32±0.03, n = 10; Figure 5A and B).

#### Study of the GABAergic Input to Locus Coeruleus Neurons of Wistar Kyoto Rats 

**Figure 6.**
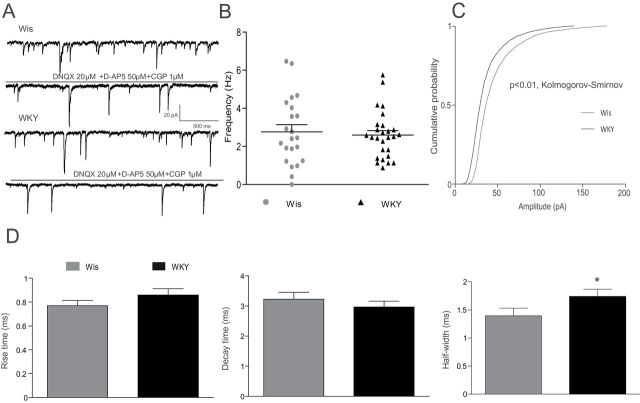
Spontaneous inhibitory input to *locus coeruleus* (LC) neurons in Wistar (Wis) and Wistar Kyoto (WKY) rats. (A) Representative current traces of spontaneous GABAergic postsynaptic currents (sIPSC) before and after pharmacological isolation. (B) Dot graphs showing the frequency of sIPSCs in LC neurons of Wis and WKY rats. (C) Cumulative histograms of sIPSCs amplitude in LC neurons of Wis and WKY rats, showing that in WKY rats the amplitude of sIPSCs was reduced (*p* < 0.01, Kolmogorov-Smirnov test). (D) Summary bar graphs of sIPSCs kinetics parameters in LC neurons, showing that half-width is greater in WKY as compared to in Wis strain (*p < 0.05, unpaired *t*-test) Bars represent the mean ± standard error of the mean of experiments (n = 22–27).

To evaluate fast inhibitory synaptic activity in LC neurons, GABA_A_ transmission was pharmacologically isolated by adding the following AMPA, NMDA, and GABA_B_ receptor blockers to the external bath solution (Figure 6A): DNQX disodium salt (20 µM), D-AP5 (50 µM), and CGP 55845 hydrochloride (1 µM). The frequency of sIPSCs in LC neurons was not statistically different between strains (Wis: 2.76±0.38 Hz, n = 22; WKY: 2.59±0.24 Hz, n = 27; Figure 6B). However, in the WKY rats the mean amplitude of sIPSCs was significantly reduced as compared to that recorded in Wis rats (Wis: 45.27±0.0.44 pA, n = 22; WKY: 34.33±0.33 pA, n = 27; *p* < 0.01, Kolmogorov-Smirnov test; Figure 6C). Regarding the kinetic of sIPSC, no differences were observed in rise time (Wis: 0.77±0.04ms, n = 18; WKY: 0.85±0.05ms, n = 25) or in decay time of the spontaneous events (Wis: 3.22±0.23ms, n = 18; WKY: 2.94±0.18ms, n = 25). However, as shown in Figure 6D, the half-width of sIPSCs in WKY rats was significantly increased compared to that recorded in Wis rats (WKY: 1.74±0.13ms, n = 18; Wis: 1.35±0.14ms, n = 25; *p* < 0.05, unpaired one-tailed *t*-test).

**Figure 7.**
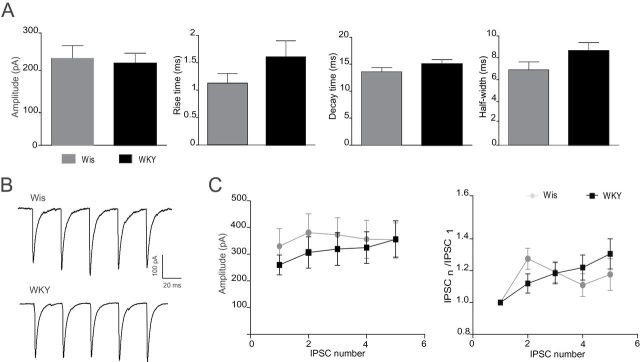
Evoked inhibitory synaptic activity in *locus coeruleus* (LC) neurons of Wistar (Wis) and Wistar Kyoto (WKY) rats. (A) Bar graphs showing the amplitude, rise time, decay time, and half-width of evoked GABAergic postsynaptic currents (eIPSCs) in LC neurons of Wis and WKY rats. (B) Representative eIPSC traces induced by a train of five pulses at 10 Hz on LC neurons of Wis and WKY rats. (C) Curve graphs showing the amplitude and IPSCn/IPSC_1_ ratio on LC neurons of Wis and WKY rats (n = 11–12). IPSCn is the amplitude of a subsequent IPSC, and IPSC1 is the amplitude of the first IPSC.

Secondly, GABA release was induced by electrical stimulation (1–10 mA) of LC neurons with a membrane potential held at -50 mV. No differences were observed in the amplitude (Wis: 231.5±34.29 pA, n = 12; WKY: 218.7±27.08 pA, n = 12) or in the parameters associated to the kinetics of eIPSC between WKY and Wis rats (rise time: 1.13±0.17ms and 1.60±0.30ms; decay time: 13.60±0.81ms and 15.02±0.86ms; and half-width: 6.89±0.73ms and 8.64±0.75ms, for Wis [n = 12] and WKY [n = 12] respectively; Figure 7A). In order to evaluate whether GABA probability release was altered in LC neurons of WKY rats, an electric stimulus train of five pulses at 10 Hz was trigged to evoke consecutive eIPSCs (Figure 7B). No statistical differences were observed in the amplitude of consecutive eIPSC or in the IPSCn/IPSC_1_ ratio between WKY and Wis rats.

It is important to note that while the frequency of inhibitory synaptic events was statistically greater in the LC of Wis rats than the frequency of the excitatory events (sIPSCs: 2.76±0.38 Hz, n = 22; sEPSCs: 1.86±0.31 Hz, n = 25; *p* < 0.05, unpaired *t*-test; see Figures 4B and 6B), in WKY rats both frequencies were similar. Interestingly, a previous patch-clamp study showed that sEPSCs frequencies were lower than sIPSCs frequencies in the LC of Sprague-Dawley rats (Sugiyama et al., 2012), in line with what we observed in the Wis strain. This suggests that synaptic activity might be dysregulated in LC neurons of WKY rats.

## Discussion

A recent *in vivo* study from our group reveals significant alterations in LC activity of the WKY rat (Bruzos-Cidon et al., 2014) in a proposed animal model of anxiety/depression (Lahmame et al., 1997; Lopez-Rubalcava and Lucki, 2000; Pare, 2000; Pardon et al., 2002; Tejani-Butt et al., 2003; De La Garza and Mahoney, 2004; Ma and Morilak, 2004; Tizabi et al., 2012). To further investigate the origin of those adaptations, the main goal of the present study was to determine whether WKY rats display a differential modulation of LC neuronal activity compared to that of Wis rats. For that purpose, intrinsic sensitivity of α_2_-adrenoceptors and extrinsic synaptic inputs to LC neurons were evaluated in Wis and WKY rats *in vitro*. Our results indicate that in LC neurons of WKY rats, α_2_-adrenoceptors are desensitized and, while spontaneous GABAergic synaptic activity is reduced, the glutamatergic activity is similar in both rat strains. The latter data may reflect an imbalance between the glutamatergic and GABAergic transmissions, which has been previously described in depressive patients (Krystal et al., 2002; Luscher et al., 2011) and WKY rats .

Regarding noradrenergic transmission in WKY rats, we have previously shown that *in vivo* LC neurons of this rat strain are less sensitive to the inhibitory effect of the α_2_-adrenoceptor agonist clonidine to the basal firing rate (Bruzos-Cidon et al., 2014). The present *in vitro* study indicates that WKY rats display lower sensitivity to α_2_-adrenoceptor agonist UK compared to the Wis strain, since the UK ratios of the peak current induced by two submaximal concentrations of the drug are significantly smaller in WKY rats compared to in Wis rats. These results confirm that noradrenergic transmission in LC neurons from WKY rats is intrinsically altered, since α_2_-adrenoceptors are desensitized *in vitro*. This could be due to a number of reasons, including both different functionality/density of α_2_-adrenoceptor and/or GIRK channels in this rat strain. Although so far expression of GIRK channels has not been investigated in the WKY rat, an early study using *in situ* hybridization showed that expression of α_2_-adrenoceptor mRNA in the LC of WKY rats is not different to that obtained in Wis rats (Sands et al., 2000). In line with this, specific binding to α_2_- adrenoceptors in the LC of WKY rats is similar to that in the control Sprague-Dawley strain (Tejani-Butt et al., 1994). All in all, dysregulation of this signaling pathway in the LC of WKY rats could be due to altered expression levels or function of other downstream components such as Gi/o proteins and/or the GIRK channels.

Evidence indicates that noradrenergic transmission is altered in WKY rats. Thus, deficient noradrenergic reactivity to acute stress (Pardon et al., 2002), low levels of NA in the LC and several of its terminal sites (De La Garza and Mahoney, 2004; Scholl et al., 2010), as well as reduced cortical NA reuptake (Jeannotte et al., 2009) have been described in this rat strain. Accordingly, it has been recently reported that acute administration of the noradrenergic antidepressant reboxetine is less potent at inhibiting *in vivo* basal activity of LC neurons in the WKY rats than that recorded in the Wis strain (Bruzos-Cidon et al., 2014). Overall, a dysregulation of the noradrenergic transmission, including the inhibitory control that α_2_-adrenoceptors exert onto neuronal activity of LC neurons, could be related to the *in vivo* LC hyperactivity observed in the WKY strain. Interestingly, chronic exposure to cold stress, which resembles alterations observed on central noradrenergic transmission of patients with mood and anxiety disorders, alters electrophysiological properties of LC neurons not only *in vivo* but also *in vitro* (Mana and Grace, 1997; Jedema and Grace, 2003).

Preclinical and clinical studies support a relevant role of the glutamatergic transmission in the pathology of psychiatric illnesses such as depression (Krystal et al., 2002; Pittenger et al., 2007). Our results show that postsynaptic sensitivity of LC neurons to glutamate is not different between the WKY and Wis strains. Regarding glutamatergic synaptic input to LC, no significant alterations have been detected in WKY rats. In the present study we separately evaluated AMPA and NMDA receptors-mediated eEPSC and we did not observe differences between the two strains. Although lower or similar NMDA receptor binding has been observed in the hippocampus, as well as in other brain areas of WKY compared to Wis rats (Lei et al., 2009), the ratio of AMPA/NMDA receptor densities is similar in both strains and is selectively increased in the WKY strain after chronic treatment with ketamine (Tizabi et al., 2012). Further experiments would be of interest to determine the electrophysiological response of noradrenergic neurons to antidepressant drugs that affect glutamatergic transmission in WKY rats. Despite lacking data about NMDA and AMPA receptor densities in LC of WKY rats, our data indicate that the synaptic activity of both NMDA and AMPA receptors is similar in the WKY and Wis strains.

The current study indicates that the frequency of sIPSC in LC neurons is similar in both strains, which suggests that release probability of GABA is not significantly altered in LC neurons of WKY strain. On the contrary, sIPSCs amplitude, which is also determined by postsynaptic factors such as receptor sensitivity, is reduced in LC neurons of WKY rats as compared with those of Wis rats. In addition, the half-width of sIPSCs is significantly greater in LC neurons of WKY rats, indicating that in this rat strain GABAergic spontaneous events are slower than in the Wis strain. Interestingly, several studies identify a GABAergic deficit as a possible cause of mood disorders. On one hand, reduced plasma and brain concentrations of GABA have been reported in depressed patients (Gerner and Hare, 1981; Petty and Sherman, 1984; Honig et al., 1988; Hasler et al., 2007). On the other, animal studies demonstrate that GABAergic transmission is dysregulated in several animal models of depression (Dennis et al., 1994; Zink et al., 2009; Veeraiah et al., 2014; Venzala et al., 2013) and that chronic administration of antidepressants markedly changes GABAergic transmission (Wabno and Hess, 2013). As previously described in Sprague-Dawley rats (Sugiyama et al., 2012), the frequency of sEPSCs and sIPSCs are uneven in LC neurons of Wis rats. Conversely, LC neurons of WKY rats showed similar frequencies of both spontaneous synaptic events, suggesting synaptic dysregulation. Since the LC spontaneous firing rate is regulated by GABAergic input (Szabo and Blier, 2001; Belujon et al., 2009), the deficient inhibitory GABAergic transmission in this brain area may be underlying the *in vivo* hyperactivity of LC neurons in WKY rats (Bruzos-Cidon et al., 2014). Corticotropin-releasing factor fails to increase GABAergic activity in dorsal raphe neurons of WKY rats, as is normally seen in Sprague-Dawley rats (Lemos et al., 2011). Altogether, these results support the GABAergic deficit hypothesis as a cause of depressive disorder in the WKY strain. Importantly, reduced GABAergic transmission in LC of WKY rats could account for the disruption of the balance between glutamatergic and GABAergic systems that has been reported as an important factor in the pathophysiology of mood disorders, including depression (Krystal et al., 2002; Luscher et al., 2011).

Our data indicate that LC neurons of WKY are differentially regulated as compared to those of Wis rats, since the noradrenergic and GABAergic systems are altered. Thus, both intrinsic and extrinsic inhibitory mechanisms are diminished in LC neurons of WKY rats. In summary, this study supports the utility of the WKY rat as a useful animal model to characterize the neurobiological bases of pathologies related to dysfunctions of multiple neurotransmitter systems, such as anxiety and depression.

## Statement of Interest

None
